# Exploring hsa_circ_0100833 as a Potential Biomarker in Oral Squamous Cell Carcinoma: Bioinformatics and Experimental Insights

**DOI:** 10.1002/cre2.70399

**Published:** 2026-07-19

**Authors:** Behnaz Raei, Yousef Seyedena, Mehrdad Hashemi, Nazanin Hosseinkhan, Mojgan Alaeddini

**Affiliations:** ^1^ Department of Genetics NT.C., Islamic Azad University Tehran Iran; ^2^ Department of Genetics TeMs.C., Islamic Azad University Tehran Iran; ^3^ Farhikhtegan Medical Convergence Sciences Research Center, Farhikhtegan Hospital, Faculty of Medicine TeMs.C., Islamic Azad University Tehran Iran; ^4^ Endocrine Research Center, The Institute of Endocrinology and Metabolism Iran University of Medical Sciences Tehran Iran; ^5^ Dental Research Center, Dentistry Research Institute Tehran University of Medical Sciences Tehran Iran

**Keywords:** biomarkers, circular RNA, oral squamous cell carcinoma

## Abstract

**Objectives:**

Oral squamous cell carcinoma (OSCC) is an aggressive malignancy with few reliable early diagnostic markers. Bioinformatic screening identified hsa_circ_0100833 as a differentially expressed circRNA in OSCC. Its potential significance in OSCC was then examined experimentally.

**Material and Methods:**

Differential circRNA expression was profiled using the GSE145608 dataset and Limma software. ROC curve analysis, circRNA–miRNA–mRNA interaction prediction, functional enrichment, and survival analysis were then used. Hsa_circ_0100833 expression was measured by qRT‐PCR in 20 paired OSCC and adjacent normal tissues. This analysis was also performed in CAL‐27 and FaDu cell lines (*n* = 3 each) and three normal human gingival fibroblast cultures. hsa‐miR‐607 and SORBS1 were quantified in parallel.

**Results:**

Differential expression analysis of the GSE145608 dataset identified hsa_circ_0100833 as down‐regulated in OSCC. Bioinformatic analysis then predicted a binding interaction between this circRNA and hsa‐miR‐607. Hsa_circ_0100833 was significantly reduced in OSCC tissues and cell lines relative to normal controls; hsa‐miR‐607 was up‐regulated, and SORBS1 showed parallel downregulation. ROC analysis revealed AUC values of 0.799 in cell lines and 0.712 in tissue samples. The predicted ceRNA network was enriched for PI3K‐Akt signaling and focal adhesion pathways.

**Conclusion:**

hsa_circ_0100833 was down‐regulated in OSCC and may act as a tumor suppressor through a hsa‐miR‐607/SORBS1‐dependent mechanism. Its diagnostic potential warrants further investigation.

## 1 Introduction

Oral squamous cell carcinoma (OSCC) is the most common malignancy of the head and neck and carries a prognosis that has changed little over recent decades (Imbesi Bellantoni et al. 2023). Most patients present at an advanced stage, and 5‐year survival remains below 60% despite multimodal therapy (surgery, radiotherapy, and chemotherapy) (Kim and Ahn 2024). A large part of this burden reflects the absence of reliable markers for early detection and treatment stratification.

Circular RNAs (circRNAs) have emerged as key post‐transcriptional regulators with well‐documented roles in cancer biology (Hussen et al. 2025). Their covalently closed structure provides considerable stability, and their tissue‐specific expression patterns point to their potential as biomarker candidates (Desouza et al. 2024). They can sequester miRNAs, thereby relieving repression of downstream target genes. This sponge activity places them within competing endogenous RNA (ceRNA) networks that regulate both oncogenic and tumor‐suppressive pathways (Mumtaz et al. 2020).

In OSCC, several well‐characterized circRNAs have already been explored, including hsa_circ_0001461, which enhances cell proliferation by acting as a miR‐145 sponge (Ai et al. 2021), and hsa_circ_0000140, which suppresses tumor growth and metastasis (Peng et al. 2020). However, most circRNAs newly detected in OSCC have not been experimentally addressed. Characterizing these molecules could expand our understanding of OSCC pathogenesis and reveal candidates with diagnostic or prognostic value. In an initial screening of OSCC transcriptomic data, we identified hsa_circ_0100833 as a differentially expressed circRNA arising from the FADS2 gene, with predicted binding sites for hsa‐miR‐607, a microRNA implicated in cancer progression. Neither hsa_circ_0100833 nor its potential interaction with hsa‑miR‑607 has been experimentally characterized in oral cancer.

The present work examined hsa_circ_0100833 expression in OSCC tissues and cell lines. Its diagnostic value and regulatory interactions within the ceRNA network were also investigated.

## 2 Materials and Methods

### 2.1 Bioinformatics Analysis

#### 2.1.1 Identification of Dysregulated circRNAs and Candidate Selection

**Figure 1:**
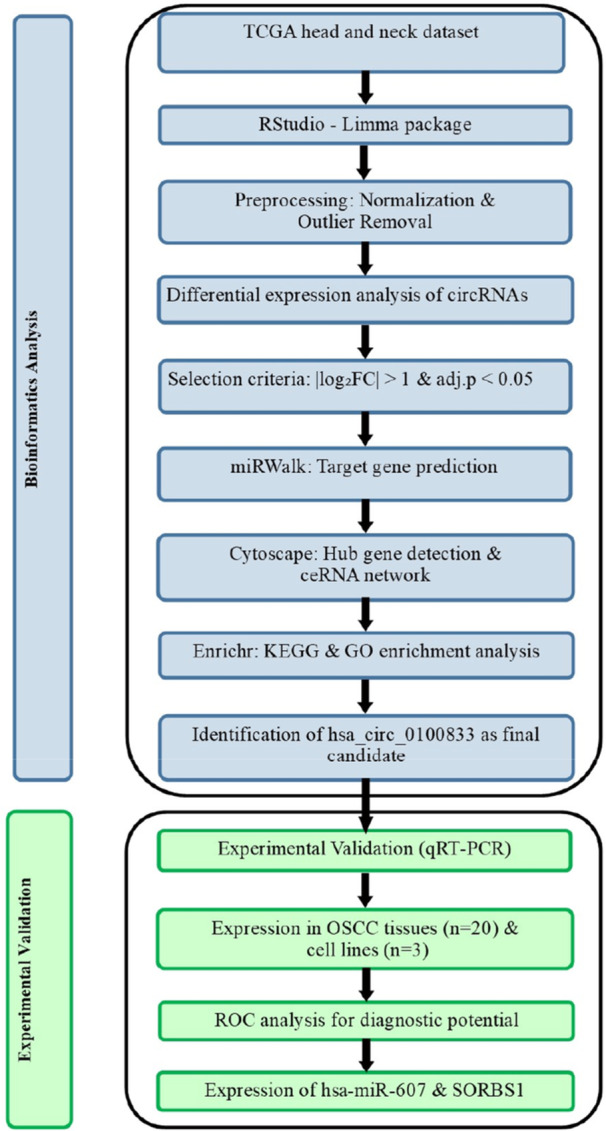
The overall workflow of the study, including bioinformatics analysis and experimental validation.

The overall workflow of the study, including bioinformatics analysis and experimental validation, is presented in Figure 1.

The pre‐normalized microarray dataset GSE145608 was retrieved from the Gene Expression Omnibus (GEO) database (https://www.ncbi.nlm.nih.gov/geo/). RStudio's Limma package (Chen et al. 2024) performed differential expression analysis on this data while using False Discovery Rate (FDR) correction for multiple testing. The selection criteria for dysregulated circRNAs required an adjusted *p*‐value below 0.05 alongside log_2_ fold change (log_2_FC) values greater than 1 for upregulation and less than −1 for downregulation.

Significantly dysregulated circRNAs received their rankings through statistical analysis that emphasized adjusted *p*‐values and absolute log_2_ fold change values. The selection of promising candidates for further research involved two criteria: (1) OSCC novel circRNA status determined by lacking specific OSCC pathogenesis connections in PubMed and Google Scholar searches, and (2) circBase database verification of circRNA identity alongside its splicing pattern and genomic coordinates. The study proceeded with experimental validation of a single circRNA candidate that ranked highest in the shortlisted candidates.

#### 2.1.2 Prediction of miRNA Response Elements (MREs)

MREs for hsa_circ_0100833 were predicted using the CircInteractome and CSCD (https://gb.whu.edu.cn/CSCD) databases. Only miRNAs with predictions overlapping in both databases were retained for downstream analysis to minimize false positives and enhance reliability (Feng et al. 2022; Panda et al. 2018; Xia et al. 2018).

#### 2.1.3 Validation of miRNA‐mRNA Interactions via miRTarBase

The interactions of the candidate miRNAs with their target mRNAs were validated using experimentally supported data from miRTarBase (Huang et al. 2020). For our candidates, we focused on interactions confirmed by methods such as reporter assays, Western blot, quantitative PCR (qPCR), next‐generation sequencing (NGS), and cross‐linking immunoprecipitation followed by sequencing (CLIP‐Seq).

#### 2.1.4 Identification of miRNA Target Genes (miTGs)

miRNA target genes were predicted using miRWalk 3.0 and TargetScan 8.0 to ensure robust initial predictions (http://mirwalk.umm.uni-heidelberg.de/) (Agarwal et al. 2015; Sticht et al. 2018). These predicted targets were further filtered using RNA‐Seq data from TCGA and the GSE13601 dataset (Estilo et al. 2009), selecting those with an adjusted *p*‐value < 0.05 and |log_2_FC| > 1, with a focus on down‐regulated genes to align with the ceRNA mechanism. Final candidates were those consistently supported across both prediction tools.

#### 2.1.5 Construction of the ceRNA Network

A circRNA–miRNA–mRNA interaction network was constructed by overlapping the predicted miRNA targets with differentially expressed genes (DEGs) from GSE13601 and The Cancer Genome Atlas (TCGA) (adjusted *p*‐value < 0.05 and |log_2_FC| > 1) using the InteractiVenn tool (https://www.interactivenn.net/). The final network was visualized using Cytoscape v3.10.0 without additional plugins (Shannon et al. 2003).

#### 2.1.6 Functional Enrichment Analysis

Kyoto Encyclopedia of Genes and Genomes (KEGG) pathway and Gene Ontology (GO) enrichment analyses were performed using Enrichr, specifically the GO Biological Process, Cellular Component, GO Molecular Function 2025, and KEGG 2021 Human libraries. Terms were considered significant if they had an adjusted *p*‐value < 0.05 after FDR correction for multiple testing (Huang et al. 2019).

#### 2.1.7 Survival Analysis

Overall survival was assessed via Kaplan–Meier analysis using UALCAN (https://ualcan.path.uab.edu/), which integrates TCGA and Genotype‐Tissue Expression (GTEx) data (Chandrashekar et al. 2022). Log‐rank *p*‐value < 0.05 was considered significant.

### 2.2 Experimental Validation

#### 2.2.1 Tissue Sampling and Cell Lines

The Ethics Committee of Islamic Azad University North Tehran Branch approved this study through IR.IAU.TNB.REC.1402.054. Twenty pairs of fresh‐frozen tissue samples derived from OSCC and adjacent non‐tumorous tissues were obtained from the Dental Research Center at Tehran University of Medical Sciences. The center had received these samples through the biobank of Imam Hospital, Tehran University of Medical Sciences. All specimens received pathological confirmation of their diagnoses, while patients remained untreated before surgery. The anatomical sites included high‐risk locations such as the tongue and floor of the mouth and lower gingiva. All tissues received immediate freezing after resection before storage at −80°C until RNA extraction occurred. The study received informed consent from every participant. The Dental Research Center provided the CAL‐27 and FaDu cell lines.

#### 2.2.2 Cell Culture

CAL27, FaDu, and HGF‐PI cells at low passages (between 5 and 10) were cultured in high‐glucose DMEM (Atocel, Austria) supplemented with 10% fetal bovine serum (FBS; Invitrogen, USA) and 1% penicillin/streptomycin (Gibco, USA). Cells were maintained at 37°C in a humidified incubator with 5% CO_2_ and routinely passaged when they reached 70%–80% confluency. For all experiments, cells were used at the specified confluency (Saberi et al. 2022).

#### 2.2.3 RNA Extraction, Complementary DNA (cDNA) Synthesis, and RT‐qPCR

Total RNA was extracted from tissue samples and cultured cells using Trizol reagent (Invitrogen, USA) according to the manufacturer's instructions. The concentration and purity of the extracted RNA were assessed using a NanoDrop 2000 spectrophotometer (Thermo Scientific, USA). cDNA was synthesized from 1 µg of total RNA using the EasyTM cDNA Synthesis Kit (ParsTous, Iran).

Quantitative real‐time PCR (RT‐qPCR) was performed using the Add SYBR Green Master Mix (high ROX, ADDBIO Inc., Korea) on a StepOnePlus Real‐Time PCR System (Applied Biosystems). The thermocycling conditions were as follows: initial denaturation at 95°C for 10 min, followed by 40 cycles of 95°C for 15 s, 60°C for 20 s, and 72°C for 30 s. Divergent primers were designed to amplify circRNAs specifically.

The primer sequences used were as follows:

- β‐actin: F: 5′‐CCTCGCCTTTGCCGATCC‐3′, R: 5′‐GGATCTTCATGAGGTAGTCAGTC‐3′ (Quan et al. 2024).
- hsa_circ_0100833: F: 5′‐TTGCCGTCATCCTTATCTTTG‐3′, R: 5′‐CTCCTACCTTCTTGCCTTATGC‐3′.

Gene expression was calculated using the comparative 2^–ΔΔCt method, with β‐actin as the endogenous control for normalization. All reactions were performed in triplicate, and data analysis was conducted using GraphPad Prism software (version 8.4.3).

The association between hsa_circ_0100833 expression levels and clinicopathological features of the 20 OSCC patients was evaluated using appropriate statistical tests. To assess the diagnostic potential of hsa_circ_0100833, receiver operating characteristic (ROC) curve analysis was performed using MedCalc software (v23.1.7). The area under the curve (AUC) was calculated to determine the ability of hsa_circ_0100833 to discriminate between OSCC and normal tissues. The optimal diagnostic cut‐off value was determined using the Youden index. The stability of the cut‐off value was internally validated via bootstrap resampling with 1000 iterations (random seed: 978). A *p*‐value less than 0.05 was considered statistically significant (Ajorlou et al. 2023).

#### 2.2.4 Expression Analysis of hsa‐miR‐607 and SORBS1 Gene

Expression data for hsa‐miR‐607 and SORBS1 in OSCC were extracted from UALCAN, comparing levels across cancer stages and sample types. For experimental validation, qRT‐PCR was conducted in CAL‐27, FaDu, and HGF‐PI cell lines. cDNA synthesis for miRNA used the mir‐Amp kit (Pars Genome, Iran), and for genes, the Easy cDNA Synthesis Kit (ParsTous, Iran). qRT‐PCR was performed with SYBR Green Master Mix (high ROX, ADDBIO Inc., Korea) under the following conditions: 95°C for 15 s, 60°C for 20 s, and 72°C for 30 s for 40 cycles. U6 snRNA and β‐actin served as internal controls for miRNA and genes, respectively. Primer sequences were as follows:

- U6: F: 5′‐GCTCGCTTCGGCAGCACATATAC‐3′, R: 5′‐CGAATTTGCGTGTCATCCTTGCG‐3′ (Mi et al. 2024).
- hsa‐miR‐607: F: 5′‐GTTCAAATCCAGATCTATAAC‐3′, R: 5′‐TGGTGTCGTGGAGTCG‐3′ (Mo et al. 2021).
- SORBS1: F: 5′‐GCATCCTTTGGAAGAGTTCG‐3′, R: 5′‐AGGACAAGTCGTCTGCAA‐3′.

Data were analyzed using GraphPad Prism (version 8.4.3). One‐way ANOVA was used for statistical comparisons, with *p* < 0.05 as significant.

## 3 Results

### 3.1 Bioinformatics Analysis

#### 3.1.1 Discovery of circRNA Involved in OSCC

**Figure 2:**
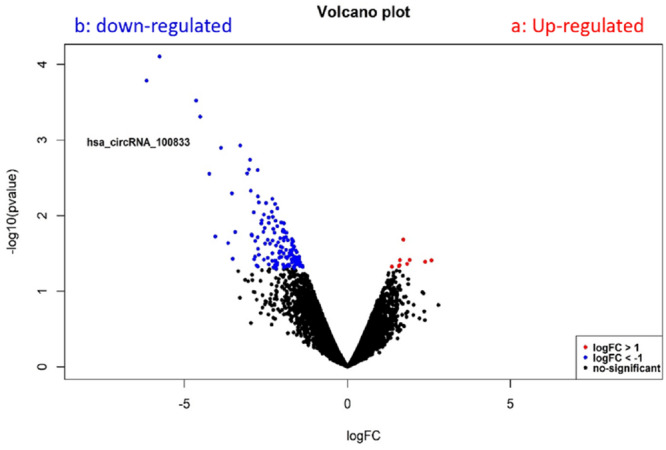
Volcano plot. Blue points represent down‐regulated circRNAs, red points represent up‐regulated circRNAs, and black points represent no significantly altered circRNAs based on *p* < 0.05 and |log_2_FC| > 1.

Differential expression analysis of circRNAs in the GSE145608 dataset revealed hsa_circ_0100833, as one of the candidate circRNAs, was significantly down‐regulated in OSCC (log_2_FC < −1, *p* < 0.05), supporting its potential involvement in OSCC pathogenesis (Figure 2).

#### 3.1.2 Prediction of miRNA Response Elements (MREs)

The results of CircInteractome and CSCD showed that four miRNAs interact with hsa_circ_0100833. All four miRNAs have been previously implicated in OSCC‐related oncogenic processes (Jun et al. 2021; Qiao et al. 2022; Saraei et al. 2021; Yao et al. 2021). The list of miRNAs and their site type is presented in Table 1.

**Table 1:** Predicted miRNA binding sites in hsa_circ_0100833.

miRNA ID	Site type	circRNA start	circRNA end
hsa‐miR‐1179	7mer‐1A	10716	10722
hsa‐miR‐607	7mer‐1A	10042	10048
hsa‐miR‐944	8mer‐1A	10213	10220
hsa‐miR‐384	8mer‐1A	1815	1822

#### 3.1.3 Prediction of miRNA Targets, KEGG Pathway, and GO Enrichment Analyses

**Figure 3:**
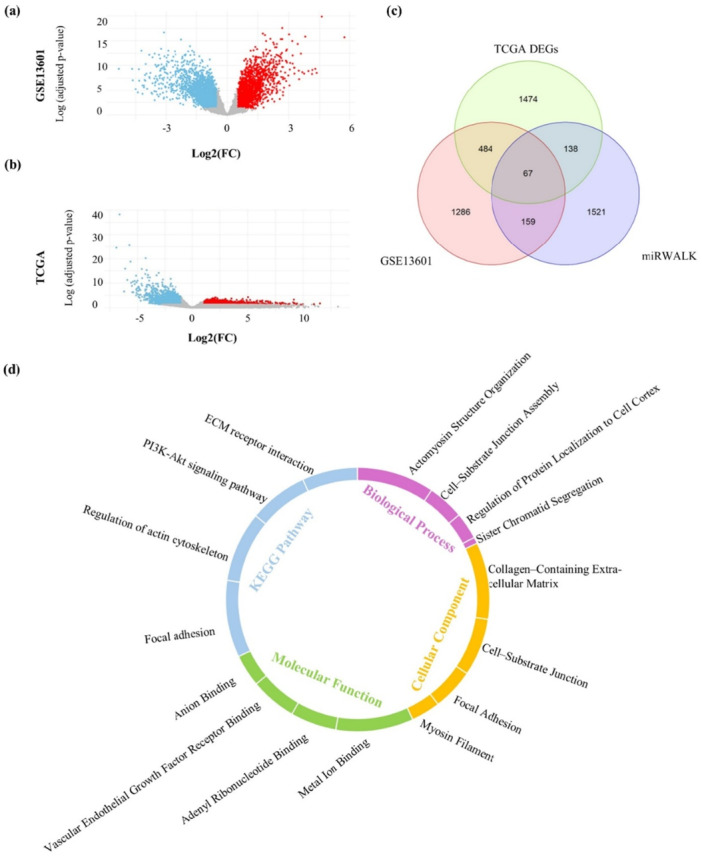
(a) A volcano plot depicting the distribution of DEGs in the GSE13601 dataset for OSCC. (b) Volcano plot of DEGs in mRNA data from TCGA. (c) A Venn diagram illustrates the overlap between DEGs and miRNA target genes. (d) A circular diagram summarizes the gene enrichment analysis of overlapping genes.

Differentially expressed genes in GSE13601 (Figure 3a) and TCGA (Figure 3b) were identified. Subsequently, an overlap analysis revealed 67 shared mRNAs among miRWalk 3.0, GSE13601, and TCGA (Figure 3c).

Functional enrichment analysis of the 67 mRNAs revealed that they are significantly involved in pathways such as the PI3K‐Akt signaling pathway, focal adhesion, regulation of the actin cytoskeleton, and ECM receptor interaction. GO analysis showed enrichment in molecular functions (e.g., metal ion binding), cellular components (e.g., collagen–containing extracellular matrix), and biological processes (e.g., actomyosin structure organization) (Figure 3d).

#### 3.1.4 Construction of the circRNA‐miRNA‐mRNA Interaction Network

An interaction network was constructed incorporating hsa_circ_0100833, the four miRNAs, and 67 mRNA targets validated by both miRTarBase and TargetScan. This network highlighted the associations between hsa_circ_0100833, four specific miRNAs, and 67 mRNAs (Figure 5a). To identify functional circRNA‐miRNA‐mRNA regulatory networks in OSCC, the degree, closeness centrality, and betweenness centrality of miRNAs were computed using the cytoHubba plugin (Cytoscape 3.4.0). The results showed that among four miRNAs, hsa‐miR‐607 had the highest degree, betweenness, and closeness (67, 0.85, and 0.911, respectively, Table S1). In the circRNA‐mRNA‐miRNA regulatory network, SORBS1 was one of the genes connected to miR‐607, which had the highest criteria and was selected for further analyses.

### 3.2 Experimental Validation

#### 3.2.1 Expression of hsa_circ_0100833 in OSCC Tissues and Cell Lines

**Figure 4:**
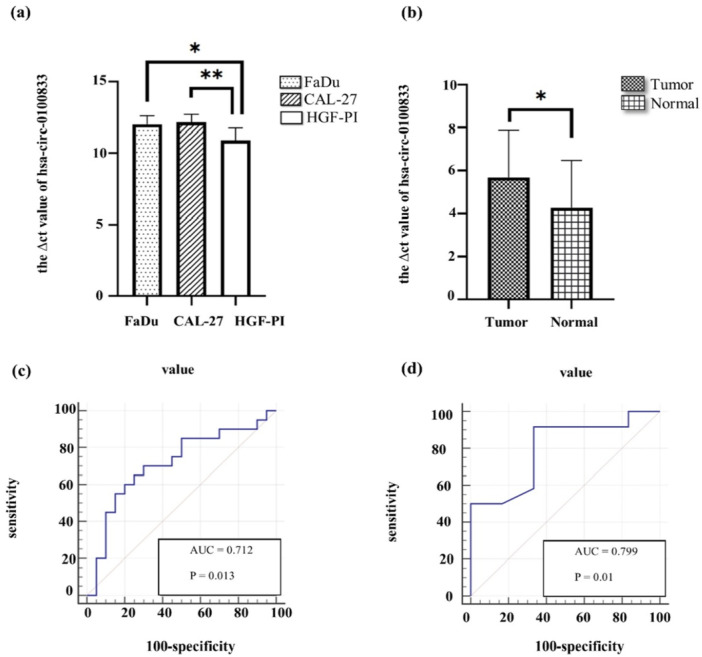
(a) hsa_circ_0100833 expression patterns (ΔCt value) in OSCC cell lines (FaDu and CAL‐27) compared to the normal cell line (HGF‐PI). The expression of hsa_circ_0100833 was significantly reduced in FaDu and CAL‐27 compared to HGF‐PI (*p* < 0.05 and *p* < 0.01, respectively). (A higher ΔCt value indicates lower expression levels). (b) The expression levels (ΔCt value) of hsa_circ_0100833 in OSCC tumor tissues were markedly lower than in normal tissues (*p* < 0.05). (c) ROC analysis was used to identify the diagnostic potential of hsa_circ_0100833 expression in 20 pairs of tissues from OSCC patients (AUC: 0.712, *p* = 0.013). (d) Diagnostic value (ROC) for the expression of hsa_circ_0100833 in cell lines (AUC:0.799, *p* = 0.01).

RT‐qPCR analysis evaluated hsa_circ_0100833 expression in 20 pairs of OSCC and their adjacent normal tissues, as well as in FaDu, CAL‐27, and the non‐tumorigenic HGF‐PI cell lines. The results showed a statistically significant downregulation in the expression of hsa_circ_0100833 in both FaDu (0.34‐fold) and CAL‐27 (0.4‐fold) cell lines compared to HGF‐PI (*p* < 0.05 and *p* < 0.01, respectively; Figure 4a). Similarly, hsa_circ_0100833 expression in OSCC tissues (0.46‐fold) was lower than in normal counterparts (*p* < 0.05; Figure 4b).

#### 3.2.2 Diagnostic and Prognostic Significance of hsa_circ_0100833

The association between hsa_circ_0100833 levels and clinicopathological features was evaluated (Table 2). Although trends were observed for tumor grade, Tumor‐Node‐Metastasis (TNM) stage, and gender, they were not statistically significant. The results demonstrated a statistically significant association between patient age and hsa_circ_0100833 (*p* = 0.02). However, the number of patients in the groups was not equal. ROC curve analysis revealed that hsa_circ_0100833 could distinguish OSCC from controls with an AUC of 0.712 (95% CI: 0.548–0.844, *p* = 0.013) in tissue samples (optimal cut‐off > 5.12, Youden Index = 0.40; sensitivity: 70%, specificity: 70%) and an AUC of 0.799 (95% CI: 0.546–0.947) in cell lines with an optimal cut‐off > 10.69 (sensitivity: 91.67%, specificity: 66.67%). Bootstrap validation (1000 iterations) confirmed these results for both tissue (AUC CI: 0.522–0.860; cut‐off CI: > 3.11– > 6.045) and cell lines (AUC CI: 0.465–0.958; cut‐off CI: > 10.11– > 11.99) (Figure 4c,d).

**Table 2:** Association between hsa_circ_0100833 expression levels and clinicopathological characteristics in OSCC patients.

Variable	Number of patients	Mean ± SD	*p* value
Tumor grade:			0.41
I–II	16	5.47 ± 2.15	
III–IV	4	6.51 ± 2.44	
TNM			0.33
I/II	10	5.92 ± 1.84	
III	10	4.71 ± 3.42	
Age			0.02*
≥ 65	7	7.35 ± 1.08	
< 65	13	4.96 ± 2.16	
Gender			0.18
F	11	6.27 ± 2.03	
M	9	4.95 ± 2.25	

* *p* < 0.05 is considered statistically significant.

#### 3.2.3 Expression Analysis of hsa‐miR‐607 and SORBS1 in OSCC Cells

**Figure 5:**
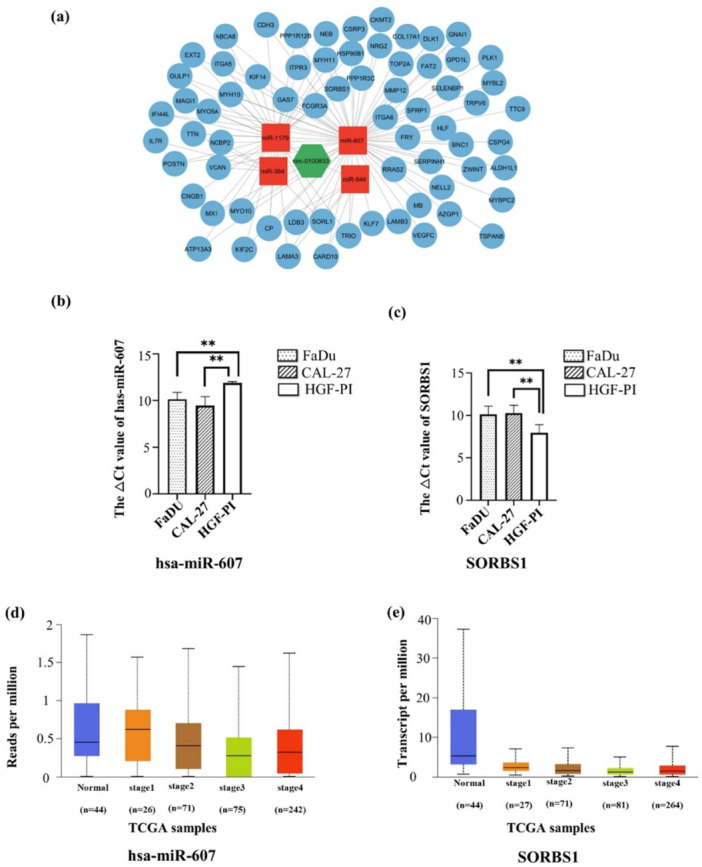
(a) Regulatory network linking circRNA, miRNA, and mRNA. Blue circles, red squares, and green hexagons refer to down‐regulated mRNAs, up‐regulated miRNAs (hsa‐miR‐607, hsa‐miR‐944, hsa‐miR‐1179, and hsa‐miR‐384), and hsa_circ_0100833, respectively. (b) The relative expression of miR‐607 in cancer cell lines (FaDu and CAL‐27) compared to the control (HGF‐PI). (c) Relative SORBS1 expression in cancer cell lines (FaDu and CAL‐27) compared to the control (HGF‐PI). (d) UALCAN results of hsa‐miR‐607 expression across various stages of HNSC. (e) UALCAN result of the expression of SORBS1 in different stages of HNSC.

The expression levels of hsa‐miR‐607 and SORBS1 were measured using relative comparative RT‐qPCR in CAL‐27 and FaDu cells, with the HGF‐PI cell line serving as a control. hsa‐miR‐607 upregulation was detected in FaDu (3.65‐fold) and CAL‐27 (6.25‐fold) cells in comparison with HGF‐PI normal cells (*p* < 0.05; Figure 5b). Conversely, SORBS1 expression levels were decreased in FaDu (0.35‐fold) and CAL‐27 (0.22‐fold) cells compared to HGF‐PI control cells (*p* < 0.05; Figure 5c). TCGA findings showed that the expression of hsa‐miR‐607 increases in the early stages of head and neck squamous cell carcinoma (HNSC) and gradually decreases (Figure 5d), while the expression of SORBS1 decreases during cancer progression (Figure 5e).

## 4 Discussion

Research in cancer biology has shown that circular RNAs (circRNAs) are important regulatory molecules because they can interact with miRNAs and influence gene expression (Chao et al. 2022). In this study, we identified hsa_circ_0100833 as a significantly down‐regulated circRNA in OSCC using comprehensive bioinformatics analysis of the GSE145608 dataset, with experimental validation supporting its decreased expression in OSCC tissues and both FaDu and CAL‐27 cell lines relative to normal controls. Notably, this decrease was observed in both the CAL‐27 cell line from the tongue and the FaDu cell line from the hypopharynx. These models are frequently used in head and neck cancer research, despite originating from different anatomical areas, which may suggest that common molecular mechanisms are at play in squamous cell carcinomas. Our results indicate that hsa_circ_0100833 may function as a tumor suppressor by sequestering hsa‐miR‐607, consequently altering the ceRNA network and suppressing target genes, such as SORBS1. hsa_circ_0100833, known as circFADS2, has been studied in several cancer types, demonstrating context‐dependent roles as either a tumor suppressor or an oncogene. In cutaneous squamous cell carcinoma, circFADS2 is down‐regulated and acts as a tumor suppressor by sequestering miR‐766‐3p, which in turn upregulates HOXA9 (Zhang et al. 2022). This inhibitory effect corresponds with our results in OSCC. In non‐small cell lung cancer, circFADS2 is increased and acts as an oncogene by sequestering miR‐498 and suppressing OMA1‐mediated mitophagy (Zhao, Han et al. 2018). In colorectal cancer, elevated circFADS2 levels are associated with larger tumor size, more advanced stages, and a worse prognosis, indicating a potential carcinogenic function via miRNA sequestration or pathway modulation (Xiao et al. 2020). The differing actions of hsa_circ_0100833 in various tumor types underscore the need for tissue‐specific research to elucidate its roles in OSCC. The predicted relationship between hsa_circ_0100833 and hsa‐miR‐607, validated through the CircInteractome and CSCD databases, highlights a ceRNA mechanism that plays a role in the progression of OSCC. Hsa‐miR‐607 showed increased levels in OSCC cell lines and early‐stage HNSC samples from TCGA (Figure 4b,d), consistent with its previously reported role in promoting cancer in other types, including pancreatic ductal adenocarcinoma, where lower serum miR‐607 levels are linked to worse outcomes and metastasis (Jiang et al. 2021). In our network analysis (Figure 4a), hsa‐miR‐607 showed the highest centrality parameters, connecting it to several mRNA targets, such as SORBS1. SORBS1, noted for its decreased levels in OSCC cells and more advanced stages of HNSC (Figure 4c,e), plays a role in cell adhesion and migration. Reduced expression has been linked to increased invasiveness in OSCC. This indicates that when hsa_circ_0100833 is down‐regulated, it allows hsa‐miR‐607 to become more active, which in turn suppresses SORBS1 and triggers pathways that promote tumor growth. The analysis of the overlapping mRNAs (Figure 3c) revealed a notable connection to PI3K‐Akt signaling, focal adhesion, and actin cytoskeleton regulation (Figure 3d), which are pathways often disrupted in OSCC (Ge et al. 2019; Veerasamy et al. 2025). The PI3K‐Akt pathway plays a crucial role in helping cells survive, develop, and prevent programmed cell death in OSCC. This pathway is often continuously activated, which is linked to a poor outcome for patients (Ghafouri‐Fard et al. 2022). Our findings suggest that the hsa_circ_0100833/miR‐607/SORBS1 axis might connect with these pathways, as shown by the volcano plots of differentially expressed genes from GSE13601 and TCGA (Figure 3a,b). Previous research indicates that circRNAs, such as circ_0072309, play a role in tumor development by influencing miR‐607 and its downstream targets in non‐small cell lung cancer (Mo et al. 2021). Therefore, focusing on this axis might provide new ways to prevent the progression of OSCC. In our investigation, hsa_circ_0100833 exhibits significant promise as a biomarker for OSCC. ROC analysis indicates its effectiveness in differentiating OSCC from control samples in both tissue and cell line studies. The use of bootstrap validation helped strengthen the reliability of these findings, even with our small group of patients. The links noted with clinicopathological features, particularly the significantly reduced expression in younger patients, suggest the increasing importance of circRNAs in the diagnosis of OSCC. Multi‐circRNA panels like hsa_circ_0001874 and hsa_circ_0001971 found in saliva have demonstrated impressive accuracy (Zhao, Wang et al. 2018). The decrease associated with age might be due to the more aggressive behavior frequently observed in OSCC in younger patients, which could involve a higher rate of metastasis, although results can differ (Hong et al. 2025). These diagnostic values suggest that hsa_circ_0100833 might improve current biomarker approaches for early detection and personalized treatment. This prospect deserves further validation in larger groups to fully confirm its prognostic significance.

The present study has some limitations. Our sample was relatively small, and group distributions were unequal, which may have influenced the clinicopathological analyses. Nonetheless, bioinformatics and qRT‐PCR evidence collectively support a role for the hsa‐circ‐0100833/hsa‐miR‐607/SORBS1 axis in OSCC pathogenesis. Studies with larger and independent patient cohorts are needed to support its diagnostic relevance and explain its role in OSCC. Furthermore, the findings depend on in vitro validation, lacking functional assays such as knockdown or overexpression to truly confirm the link between variables. Also, our standard control cell line (HGF‐PI) was obtained from human gingival fibroblasts rather than keratinocytes. While this choice may not completely reflect the characteristics of normal oral epithelium, it is worth noting that similar fibroblast controls have been used in other studies on OSCC (di Giacomo et al. 2025; Ringer et al. 2020). Future research could benefit from exploring in vivo models, incorporating multiple omics, and conducting longitudinal studies to evaluate the therapeutic targeting of hsa_circ_0100833.

## 5 Conclusion

Our data suggest that hsa_circ_0100833 is down‐regulated in OSCC and may function as a tumor suppressor through the miR‐607/SORBS1 axis. These findings support its further evaluation as a diagnostic biomarker and potential therapeutic target.

## Author Contributions

Conception and design: Mehrdad Hashemi and Mojgan Alaeddini. Provision of study: Mehrdad Hashemi, Behnaz Raei, and Nazanin Hosseinkhan. Collection and assembly of data: Behnaz Raei, Nazanin Hosseinkhan, Yousef Seyedena, and Mehrdad Hashemi. Data analysis and interpretation: Behnaz Raei, Mojgan Alaeddini, and Mehrdad Hashemi. Manuscript writing and final approval of manuscript: all authors.

## Funding

The authors have nothing to report.

## Ethics Statement

All experiments were conducted in accordance with relevant guidelines and regulations (Helsinki Declaration) and the study was approved by the Ethics Committee of NT.C., Islamic Azad University, Tehran, Iran (Ethical code: IR.IAU.TNB.REC.1402.054). This study is not clinical trials.

## Consent

Written informed consent was obtained from all participants, and confidentiality and anonymization of patient data were strictly maintained.

## Conflicts of Interest

The authors declare no conflicts of interest.

## Supporting information


**Supporting File 1:** cre270399‐sup‐0001‐supplementary_link.docx.

**Supporting File 2:** cre270399‐sup‐0002‐Table_1.docx.

## Data Availability

All relevant data and materials are included within the manuscript. Any additional data supporting the findings of this study are available from the corresponding authors upon reasonable request.
