# Supplementary material for: Exploring hsa_circ_0100833 as a Potential Biomarker in Oral Squamous Cell Carcinoma: Bioinformatics and Experimental Insights
Source: Clin Exp Dent Res. 2026 Jul 19;12(4):e70399. doi: 10.1002/cre2.70399 (PMC13380815; doi:10.1002/cre2.70399)
Supplement: Supplementary file 1 — Supporting File 1: cre270399‐sup‐0001‐supplementary_link.docx. [file CRE2-12-e70399-s002.docx]

<https://figshare.com/s/469a734e3377f25c017c>
