# Supplementary material for: Exploring hsa_circ_0100833 as a Potential Biomarker in Oral Squamous Cell Carcinoma: Bioinformatics and Experimental Insights
Source: Clin Exp Dent Res. 2026 Jul 19;12(4):e70399. doi: 10.1002/cre2.70399 (PMC13380815; doi:10.1002/cre2.70399)
Supplement: Supplementary file 2 — Supporting File 2: cre270399‐sup‐0002‐Table_1.docx. [file CRE2-12-e70399-s001.docx]

**Table 1.** degree, betweenness, and closeness for hsa-miR-607, hsa-miR-1179, hsa-miR-384, hsa-miR-944

| name | Degree | BetweennessCentrality | ClosenessCentrality |
| --- | --- | --- | --- |
| hsa-miR-607 | 67 | 0.84594 | 0.911392 |
| hsa-miR-1179 | 33 | 0.13683 | 0.489796 |
| hsa-miR-384 | 8 | 0.031286 | 0.365482 |
| hsa-miR-944 | 7 | 0.004333 | 0.361809 |
